# The role of pregnancy acceptability in maternal mental health and bonding during pregnancy

**DOI:** 10.1186/s12884-022-04558-6

**Published:** 2022-03-29

**Authors:** Josephine McNamara, Alixandra Risi, Amy L. Bird, Michelle L. Townsend, Jane S. Herbert

**Affiliations:** 1 School of Psychology, University of Wollongong, Wollongong, NSW 2522 Australia; 2 Early Start, University of Wollongong, Wollongong, NSW 2522 Australia; 3 School of Psychology, University of Waikato, Hamilton, Waikato 3240 New Zealand; 4 Illawarra Health and Medical Research Institute, University of Wollongong, Wollongong, NSW 2522 Australia

**Keywords:** Mental health, Maternal–fetal attachment, Antenatal bonding, Pregnancy acceptability, Pregnancy intendedness

## Abstract

**Background:**

Pregnancy is an important time for women’s mental health and marks the foundations of the emerging bond between mother and baby. This study aimed to investigate the role of pregnancy acceptability and intendedness in maternal mental health and bonding during pregnancy.

**Methods:**

Data were collected from a community sample of 116 Australian pregnant women (*M* = 29.54, *SD* = 5.31) through a series of self-report questionnaires pertaining to mental health and antenatal bonding.

**Results:**

Lower pregnancy acceptability was correlated with higher depression, anxiety and total distress, lower physical and environmental quality of life and lower antenatal bonding. Women who reported their pregnancy was intended reported higher physical quality of life than those who reported their pregnancy was unintended. The relationship between total distress and antenatal bonding was moderated by women’s degree of pregnancy acceptability (low versus high). For women with low acceptability, higher distress was associated with lower bonding, but there was no such association for women with high pregnancy acceptability. The moderation model examining associations between distress and pregnancy acceptability explained 15% of the variance in antenatal bonding scores.

**Conclusion:**

Consideration of women’s appraisal of their pregnancy acceptability may provide a valuable framework for identifying individuals who may be at risk for mental health and bonding difficulties.

## Introduction

Pregnancy marks a period of emotional, physical, identity and relational changes that are largely shaped by women’s individual circumstances [1]. For some women, learning of a pregnancy is an overwhelmingly positive experience. For others, it may evoke fear and anguish; or feelings of shock, surprise or ambivalence [2, 3]. Pregnancy acceptability is a term used to describe how a woman thinks and feels about a pregnancy once she learns of it [4]. The concept of pregnancy acceptability aims to capture a woman’s appraisal of the desirability and timing of the pregnancy after conception [5]. Previous frameworks have used a pregnancy intendedness model, founded on a planned versus unplanned dichotomy, to identify women at risk of mental health and early bonding difficulties [6]. Given the complex trajectory of pregnancy emotions and experiences, a dichotomy based on initial reproductive intentions may be limited for understanding and supporting pregnant women [7, 8]. In this article, we explore whether a woman’s response to pregnancy, that is, her assessment of pregnancy acceptability, is associated with maternal mental health and bonding during pregnancy.

The pregnancy intendedness model holds that pregnancy can be categorised as intended, mistimed or unwanted, with the latter two groups forming an umbrella category of ‘unintended’ pregnancy [9]. International research suggests that approximately half of all pregnancies [10], and 40% of pregnancies that are continued to birth, are unintended [11–13]. Unintended pregnancy is associated with delayed antenatal care and fewer health-related behaviours during pregnancy for mothers [14, 15], as well as increased risk of need for neonatal special care after birth, breast-feeding difficulties [16], and mental health and behavioural problems in children [17]. For these reasons, the intended or unintended nature of women’s pregnancies has been an area of sustained research attention over the last 20 years. Some studies have found that women with unintended pregnancies find it more difficult to establish a bond with their baby [18, 19] and maintain good mental health [14, 15] during pregnancy, however other studies have not found significant results [20–22].

Although the straightforwardness of the intended versus unintended pregnancy dichotomy is valuable, it has also been subject to criticism for over-simplifying the complexities of pregnancy [23, 24]. The intendedness model requires assumptions to be made about women’s reproductive decisions when planning does not occur and does not account for circumstances in which a pregnancy may not be planned but welcomed [8]. In particular, the model may be insufficiently sensitive to individual differences in women’s attitudes towards their pregnancy [7] as it does not account for feelings of ambivalence often reported by women [24, 25] and the fact that many women report varying attitudes towards intendedness throughout their pregnancy [26]. Awareness of these limitations has prompted a reconsideration of whether pregnancy intendedness provides a sound basis for clinical decisions in identifying women in need of support [27]. One concept that has emerged to address this gap is pregnancy acceptability [4].

Pregnancy acceptability is defined as the degree to which women consider their pregnancy ‘acceptable’ after conception [28]. It takes into account a women’s appraisal of the desirability and timing of the pregnancy [5], the congruence of pregnancy intentions and fertility-related behaviours [24] and the range of emotions experienced when she learns of the pregnancy [24]. The pregnancy acceptability framework acknowledges that a woman’s intentions and feelings towards her pregnancy may be multi-dimensional and incongruent [29, 30]. This aspect of the model is supported by empirical research which suggests that 68% of women describe their unintended pregnancy as “wanted” [31] and that women report rewarding parts of unintended pregnancy such as improvement in partner relationship, recognition of resilience and avoiding waiting for the “perfect time” to have a baby [32]. A recent study found that couples based their pregnancy acceptability on factors such as relationship stability, feeling prepared to and capable of being a parent and taking a flexible approach towards family planning [7]. These studies highlight the value of understanding women’s cognitive and emotional responses to pregnancy. They suggest that the way a woman feels in response to learning of a pregnancy may impact upon the way she feels towards herself, her baby and the emotional bond that develops between the dyad.

The emotional bond between a mother and her infant begins during pregnancy and marks the origins of the mother-infant relationship and the foundation for future interactions [33]. Antenatal bonding, initially described as maternal fetal attachment, was introduced by Cranley [34] to describe the behaviors pregnant women engaged in that marked a desire to interact with and form a relationship with their unborn child. Antenatal bonding exclusively focuses on the affective tie from mother to baby [35, 36] and is made up of thoughts, behaviours and feelings [37, 38]. Approximately 10–15% of women do not develop a bond towards their baby by the third trimester [39]. Bonding impairment appears stable across the antenatal and postnatal periods [35, 40] and predicts lower responsive and sensitive parenting [41], insecure mother-infant attachment [42] and mental health problems in children [43]. Therefore, it is important to understand whether low pregnancy acceptability may inhibit antenatal bonding.

Developing an emotional connection to one’s baby may prove to be particularly challenging for the one in five women who experience mental health difficulties from conception to one year postpartum [44]. A study by McConachie and colleagues [45] found that 40% of women rated their wellbeing as poor during the transition to motherhood. This is especially significant because poor mental health during pregnancy is associated with impaired antenatal bonding [18, 39] perhaps due to a lack of emotional resources, beliefs about poor parenting competency, and negative attitudes towards caregiving [46, 47]. Depression has consistently been shown to be associated with lower antenatal bonding [48, 49]. Anxiety has been found to be negatively associated with antenatal bonding quality, while inconsistent findings have been reported in relation to antenatal bonding as a global construct [50, 51]. A small number of studies have found that women with higher stress [52, 53], lower subjective wellbeing [54] and positive affect [55, 56] report lower antenatal bonding. To date, no studies have been conducted to examine the potential role of pregnancy acceptability in maternal mental health and antenatal bonding. It may be that pregnancy acceptability can help to explain the relationship between maternal mental health and antenatal bonding. If women with low pregnancy acceptability are more vulnerable to the stressors of pregnancy, we might expect to see an association between distress and bonding for these women in particular.

The period following confirmation of pregnancy represents a significant transition period and is likely to involve an appraisal of a wide range of factors including desirability, suitability of timing, implications for identity, achievement of goals and alignment with values. Being able to capture the way women think and feel about their pregnancy, in addition to understanding their pregnancy intentions, may be useful in supporting women’s mental health and early mother-to-baby bonding. In this paper, we examined the role of pregnancy intendedness and acceptability in mental health and bonding during early pregnancy in a community sample of Australian women. We hypothesised that women with low pregnancy acceptability would report higher distress, lower wellbeing and lower antenatal bonding. We also explored whether pregnancy acceptability moderates an association between maternal distress and antenatal bonding, but given a lack of existing research, no specific hypotheses were made.

## Methods

### Design and procedure

This study comprised part of the first wave of data collection for a larger project entitled ‘Maternal Wellbeing and Bonding.’ Participants in the larger study were asked to complete a series of questionnaires pertaining to mental health and bonding, a survey about their pregnancy experiences and a brief phone interview. The current study utilized a cross-sectional design where women completed self-report questionnaires in early pregnancy from June to October 2018. Ethical approval for this study was granted through the University of Wollongong Human Research Ethics Committee (reference: 2017/277) and hospital site specific assessment.

### Participants

Participants were 116 pregnant women receiving outpatient care at a public antenatal clinic in New South Wales [57] who were in their first or second trimester of a singleton pregnancy, 18 years or over and English-speaking. Eligible women were provided with a summary of the research aims when they arrived for their scheduled antenatal appointment and were invited to participate in the study by the first author. Recruitment took place at Wollongong Hospital Antenatal Clinic located in New South Wales, Australia which is a large regional hospital providing generalist and specialist maternity services to women across a catchment area of 250 km [58]. A total of 122 women provided consent to participate in the study, however six participants were excluded due to non-completion of greater than 25% of measures.

### Measures

#### Demographics

Women completed a demographic information questionnaire including questions about their ethnicity, age, education, relationship status, current pregnancy and previous pregnancy history.

#### Pregnancy intendedness and acceptability

To assess pregnancy intendedness, women were asked to report if their pregnancy was planned or unplanned. For unplanned pregnancy, women were asked to report their feelings about the pregnancy by selecting one of four response options: 1) “I was pleased about the pregnancy virtually from the start;” 2) “I had mixed feeling initially, but am now pleased about it;” 3) “I still have mixed feelings;” and 4) “I am mostly not happy about the pregnancy.” Based on their responses to the pregnancy intendedness and response to unplanned pregnancy questions, participants were categorised into one of two groups: 1) *high* pregnancy acceptability – women with intended pregnancy and women with unintended pregnancy who reported being pleased about the pregnancy from the start; and 2) *low* pregnancy acceptability – women with an unintended pregnancy who reported ambivalent or negative feelings towards the pregnancy.

##### World Health Organisation Quality of Life Scale (WHOQOL-Bref)

WHOQOL-Bref is a 26-item questionnaire measuring physical, psychological, social and environmental quality of life (QOL). It has been validated for use in postpartum [59] and used in other pregnancy studies [60]. WHOQOL-Bref has good reliability and internal consistency [61], and exhibited a high level of internal consistency in the current study (Cronbach’s alpha = 0.89).

##### Depression, Anxiety and Stress Scale (DASS-21)

DASS-21 is a 21-item questionnaire that assesses symptoms of depression, anxiety and stress [62] and has been validated for use in perinatal populations [63]. The DASS-21 total score was used in the current study as it has been found to be an appropriate measure of general psychological distress [64, 65]. DASS-21 shows high reliability and internal consistency [66] and exhibited a high level of internal consistency in the current study (Cronbach’s alpha = 0.89).

##### Maternal Fetal Attachment Scale (MFAS)

MFAS is a 24-item self-report questionnaire that assesses the extent to which women engage in behaviors that represent an affiliation towards their unborn child [34]. MFAS includes five subscales: 1) *differentiation* of self from fetus; 2) *interaction* with fetus; 3) *characteristics* and intentions to fetus; 4) *giving* of self; and 5) *role taking*. There is empirical support for interpreting subscale [67] and total scores for research purposes [37]. MFAS shows good reliability and internal consistency [68] and exhibited a high level of internal consistency in the current study (Cronbach’s alpha = 0.79).

#### Statistical analysis

Statistical analyses were conducted using the Statistical Package for the Social Sciences (SPSS for Windows, Version 23), and Hayes’ [69] PROCESS macro for SPSS. Data screening and cleaning was conducted prior to analysis. Expectation maximisation was used to impute missing cases for continuous variables (4%). A missing values analysis indicated that Little’s (1988) test of Missing Completely at Random (MCAR) was not significant: χ^2^ 1.44, *DF* = 3, *p* = 0.696*.* WHOQOL and MFAS scores were normally distributed. DASS-21 scores were positively skewed and were transformed with square root transformations for correlation analyses. Pearson correlation coefficients were calculated to examine associations between mental health, bonding, pregnancy and demographic variables. Alpha values smaller than 0.05 were considered significant. A MANOVA was performed to calculate potential differences in women based on pregnancy intendedness and acceptability. Moderation modelling with bootstrapping was conducted to examine the relationship between mental health, antenatal bonding and pregnancy acceptability. The PROCESS macro [70] was chosen for use because of its suitability for non-normal and asymmetrical distributions, and to balance power and validity concerns [71, 72]. For the moderation model, the bias-corrected bootstrap confidence intervals for each of the indirect effects were based on 5000 bootstrap samples using 95% confidence intervals. The indirect pathway was supported when the confidence intervals did not cross zero.

## Results

### Participant demographics

Women were aged 18–41 years (*M* = 29.54, *SD* = 5.31) with a mean gestational age of 18.78 weeks (*SD* = 4.37, range 12–27 weeks). Most women were married or in a de facto relationship (87.9%), born in Australia (90.5%), and identified English as their first language (94.0%). Six women (5.3%) identified as being of Aboriginal and/or Torres Strait Islander descent. Most women were working either full-time (36.3%) or part-time (36.3%). Annual household income ranged from < $20,000 to > $160,000 (median bracket – AUS$80,000-$100,000). Maternal education ranged from completing Years 7–9 (4.3%), Year 10 (12.9%), Year 12 (6.9%), vocational education (40.5%) and university (35.3%). Of the women, 21.6% were nulliparous, with the remaining women having between 1–9 children (*M* = 1.21, *SD* = 1.41). Almost half of the women (44%) had experienced at least one previous miscarriage (range = 0–4, *M* = 0.89, *SD* = 0.50). Regarding pregnancy intendedness, 60.3% of women (*n* = 70) reported their pregnancy was intended and the remaining 39.7% (*n* = 46) stated their pregnancy was unintended. Regarding pregnancy acceptability, 73.3% of women fell within the *high* pregnancy acceptability group (*n* = 85) and 26.7% fell within the *low* pregnancy acceptability group (*n* = 31).

### Preliminary analyses

Pearson correlation coefficients were calculated between all subscale scores for maternal mental health and bonding. Social QOL was positively correlated with MFAS-Total (*r* = 0.191, *p* = 0.040) and MFAS-Role-taking (*r* = 0.193, *p* = 0.037). Depression was negatively correlated with MFAS-Characteristics (*r* = -0.190, *p* = 0.042). Stress was negatively correlated with MFAS-Total (*r* = -0.194, *p* = 0.036) and MFAS-Characteristics (*r* = -0.196, *p* = 0.035). No other statistically significant correlations were found between mental health and antenatal bonding variables. Significant correlations between demographic variables, mental health and antenatal bonding are reported in Table 1.

**Table 1:** Significant correlations between demographic variables and measures of maternal mental health and antenatal bonding

Demographic variable	Mental health and bonding variable	*r*	*p*
Pregnancy intendedness	WHO-Physical	0.268	0.004
Pregnancy acceptability	WHO-Physical	0.273	0.003
	WHO-Environmental	0.195	0.036
	DASS-Total	-0.226	0.015
	DASS-Depression	-0.260	0.005
	DASS-Anxiety	-0.235	0.011
	MFAS-Total	0.214	0.021
	MFAS-Characteristics	0.197	0.034
	MFAS-Giving	0.214	0.021
Gestational age	MFAS-Differentiation	0.306	0.001
	MFAS-Characteristics	0.191	0.040
Fertility treatment	WHO-Physical	0.186	0.047
Parity	WHO-Physical	-0.226	0.015
Age	WHO-Stress	-0.204	0.029
Miscarriage	WHO-Physical	-0.287	0.025
	MFAS-Total	0.190	0.044
	MFAS-Giving	0.307	0.001

Results of the first MANOVA indicated a statistically significant difference in women’s mental health and antenatal bonding based on pregnancy intendedness, *F*(12, 103) = 2.088, *p* = 0.024; *Wilk’s* Λ = 0.804. Women with unintended pregnancy (*M* = 69.93, *SD* = 16.63) reported significantly poorer physical QOL than women with intended pregnancy (*M* = 78.56, *SD* = 14.22) (*p* = 0.004). No other statistically significant differences were found in relation to mental health or antenatal bonding variables. Independent sample t-tests revealed that women with unintended pregnancy had a higher gestational age (*p* = 0.036), more children (*p* = 0.010), were less likely to be married or in a de facto relationship (*p* = 0.001), had lower income (*p* = 0.026) and held fewer educational qualifications (*p* = 0.000).

Results of the second MANOVA indicated a statistically significant difference in women’s mental health and antenatal bonding based on pregnancy acceptability, *F*(12, 103) = 2.121, *p* < 0.05; *Wilk’s* Λ = 0.802. Women with low pregnancy acceptability reported significantly lower physical and environmental QOL, and higher depression, anxiety and total distress compared with women with high pregnancy acceptability. Women with low pregnancy acceptability showed lower psychological QOL than high pregnancy acceptability women, however this was not statistically significantly different. No differences were found for social QOL or stress. In relation to antenatal bonding, MFAS-Total, MFAS-Characteristics and MFAS-Giving scores were greater in women with high pregnancy acceptability (see Table 2 for further details). Women with low pregnancy acceptability had a higher number of children (*p* = 0.014) and people living in their home (*p* = 0.011), were less likely to be married or in a de facto relationship (*p* = 0.004), and held fewer educational qualifications (*p* = 0.003).

**Table 2:** Group differences across maternal mental health and wellbeing measures for low and high pregnancy acceptability groups

Variable	Acceptability	*M*	*SD*	*F*	*p*
WHO-Physical	Low	68.03	17.15	9.172	0.003
	High	77.73	14.53		
WHO-Psychological	Low	73.71	11.98	3.854	0.052
	High	78.59	11.80		
WHO-Social	Low	78.81	16.09	1.093	0.298
	High	81.98	13.82		
WHO-Environmental	Low	78.97	13.17	4.525	0.036
	High	84.86	13.21		
DASS-Depression	Low	1.36	1.03	10.201	0.002
	High	0.83	0.81		
DASS-Anxiety	Low	1.76	0.85	5.558	0.020
	High	1.27	0.91		
DASS-Stress	Low	2.03	1.11	2.419	0.123
	High	1.82	0.93		
DASS-Total	Low	3.20	1.36	6.650	0.011
	High	2.54	1.23		
MFAS-Total	Low	82.75	13.07	5.480	0.021
	High	88.03	9.77		
MFAS-Differentiation	Low	15.81	2.66	2.234	0.138
	High	16.49	2.00		
MFAS-Interaction	Low	16.35	3.33	2.147	0.146
	High	17.29	2.95		
MFAS-Characteristics	Low	20.08	4.03	4.582	0.034
	High	21.70	3.44		
MFAS-Giving	Low	14.51	3.74	5.490	0.021
	High	15.76	1.96		
MFAS-Role-taking	Low	16.00	2.94	2.047	0.155
	High	16.78	2.45		

### Main analyses

**Fig. 1:**
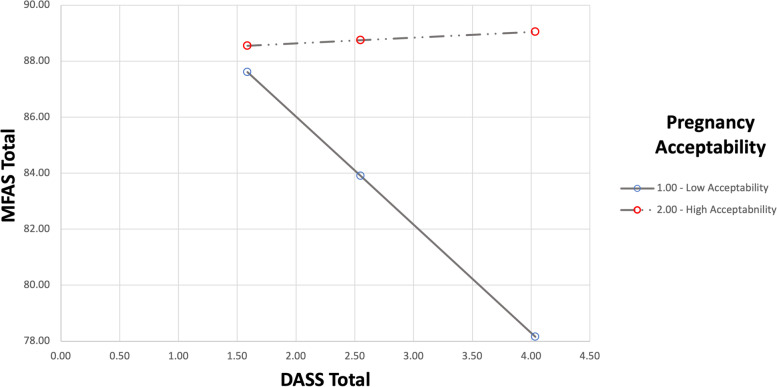
Graphical representation of focal predictor at values of the moderator (pregnancy acceptability)

The association between mental health, antenatal bonding and pregnancy acceptability was further explored through moderation analysis. MFAS-total was entered as the dependent variable, DASS-total as the independent variable and pregnancy acceptability as the predicted moderator. Based on preliminary analyses, history of miscarriage, relationship status (married or de facto versus separated or single) and educational qualifications (university educated versus high school or trade qualification) were entered as covariates. The model explained 15.06% of the variance in antenatal bonding: *F*(6, 106) = 3.13, *p* = 0.0073, *R*^*2*^ = 0.1506. Unstandardized coefficients, SEs, and 95% CIs are shown in Table 3. DASS-total was a significant individual predictor of antenatal bonding: *B* = -6.97, *t*(106) = -3.00, *p* = 0.0219, but pregnancy acceptability was not: *B* = -4.07, *t*(106) = -0.73, *p* = 0.4680. The interaction effect was statistically significant and different from zero: *B* = 3.48, *t*(106) = 2.04, *p* = 0.0434, indicating that the association of distress with antenatal bonding depends on women’s degree of pregnancy acceptability. In the low acceptability group, there was a statistically significant relationship between antenatal bonding and distress: *B* = -3.49, *t*(106) = -2.45, *p* = 0.0160, 95% CI [-6.32, -0.66]. For the high acceptability group, no statistically significant relationship existed between antenatal bonding and distress: *B* = -0.02, *t*(106) = -0.02, *p* = 0.9863, 95% CI [-1.88, 1.85] (see Fig. 1). These findings indicate that pregnancy acceptability impacted on distress and bonding for women who reported ambivalent or negative feelings towards their pregnancy (low acceptability) but not those who reported entirely positive feelings (high acceptability). Additional models with social and psychological QOL as the independent variables were tested, but were non-significant.

**Table 3:** Model coefficients for testing moderation of the relationship between antenatal bonding and distress by pregnancy acceptability

	*B*	SE	*t*	*p*	LLCI	ULCI
DASS-total	-6.97	3.00	-2.33	0.0219	-12.91	-1.03
Acceptability	-4.07	5.59	-0.73	0.4680	-15.15	7.01
Covariate (miscarriage)	4.08	2.01	2.03	0.0448	0.10	8.06
Covariate (relationship status)	-3.83	3.31	-1.16	0.2494	-10.39	2.73
Covariate (education)	-2.71	2.17	-1.25	0.2140	-7.02	1.59
Acceptability x DASS-total	3.48	1.70	2.04	0.0434	0.11	6.85
Constant	99.32	10.47	9.49	0.000	78.57	120.07

*R*^2^ 0.1506, *F*(6, 106) 3.13, *p* 0.0073

## Discussion

In this study we investigated the role of pregnancy intendedness and acceptability in maternal mental health and antenatal bonding in a sample of 116 Australian pregnant women. Domains of antenatal bonding were positively correlated with social QOL and negatively correlated with depression and stress, but not other domains of QOL or anxiety. Group differences emerged between the high and low pregnancy acceptability groups indicating poorer mental health and bonding for women with lower pregnancy acceptability. After controlling for a number of socio-demographic covariates, pregnancy acceptability moderated the relationship between overall distress and antenatal bonding.

### Pregnancy intendedness

Consistent with previous research [73], we found that women with unintended pregnancy reported poorer physical QOL than women with intended pregnancy. This finding suggests that women who reported their pregnancy as unintended endorsed reduced mobility and access to services, and poorer satisfaction with sleep and their ability to work and engage in activities. Puente and colleagues [74] suggested that a reduced locus of control experienced when a pregnancy is not planned may affect a woman’s appraisal of common pregnancy symptoms (e.g., nausea, vomiting) and increase the impact of these symptoms on her functioning. We found no other group differences based on pregnancy intendedness in relation to mental health or bonding, supporting our hypothesis that the pregnancy intendedness model may not provide a sensitive enough framework by which to identify women in need of support. Similar patterns relating to demographic variables emerged between the pregnancy intendedness and acceptability groups. Women with unintended pregnancy and low pregnancy acceptability reported having had a higher number of children, were less likely to be married or in a de facto relationship and held fewer educational qualifications. This finding was not unexpected given that all of the women in the low acceptability group classified their pregnancy as unintended. However, it suggests that previous research examining intendedness may have unknowingly tapped into the acceptability construct. This potential explanation is of course speculative and requires future longitudinal research tracking pregnancy intendedness and acceptability before and during pregnancy.

### Using pregnancy acceptability as a framework

When our sample was analysed based on pregnancy acceptability, a number of group differences emerged. Compared with the high acceptability group, women with low pregnancy acceptability reported significantly lower physical and environmental QOL, and higher depression, anxiety and total distress. Women with low pregnancy acceptability reported lower global antenatal bonding, in addition to lower scores on the *Characteristics* and *Role-taking* subscales of the MFAS. These findings suggest that regardless of whether the pregnancy was intended, a woman’s cognitive and emotional appraisal of her pregnancy is related to the way she feels about herself and her baby. For women who reported ambivalent or mostly negative feelings towards their pregnancy (low acceptability), their evaluation may have reflected poor timing and desirability of the pregnancy based on current circumstances and future goals and a disconnect between reality and intentions around fertility behaviour [24]. Adjusting to the idea of pregnancy and parenthood may have involved unexpected changes in career trajectory, stress about financial stability, questioning of relationship status and ambivalence around readiness and preparedness for parenting [7]. The cross-sectional nature of our data means that we cannot infer causation about the nature of this relationship. Our data may indicate that low pregnancy acceptability led to an increase in distress, decrease in quality of life and poorer antenatal bonding. Alternatively, women’s existing mental health and emotional connection towards their baby may have contributed to their appraisal of the acceptability of the pregnancy.

### Pregnancy acceptability in mental health and bonding

Further analyses showed that the association of distress with antenatal bonding was dependent on women’s appraisal of pregnancy acceptability. Our moderation model highlighted a relationship between psychological distress and antenatal bonding for women who reported ambivalent or negative feelings (low acceptability) but not those who reported entirely positive feelings towards their pregnancy (high acceptability). This suggests that a woman who experiences ambivalent or negative feelings towards her pregnancy and symptoms of psychological distress (e.g., depression, anxiety, stress), may find it more difficult to form positive mental representations of her baby and engage in behaviors that signify a desire for closeness and interaction with her baby. This is an important consideration during the antenatal period as women with low pregnancy acceptability, who are also experiencing psychological distress, appear to be at increased risk of antenatal bonding difficulties.

### Strengths and limitations

A strength of this study was the diverse group of women who participated. They came from a range of backgrounds and had diverse pregnancy histories. Participant diversity was facilitated by the demographic profile of the hospital at which recruitment took place. It is the largest in the region, supports a 250 km catchment area, provides generalist and specialist maternity services and offers a range of antenatal care options for women. A methodological limitation of this study was that we only asked women with unintended pregnancy about their response to their pregnancy. While we assume that women with planned pregnancies experience a high degree of pregnancy acceptability, it would have been preferential to ask all women about their response. Future research would benefit from asking all women about their feelings towards their pregnancy regardless of intendedness and seeking to measure pregnancy intendedness and acceptability in more nuanced ways. Asking women about the way their partners felt about the pregnancy may also play a role in women’s appraisal of pregnancy acceptability and would a fruitful area for future research. Future studies could also investigate the role that social support from family and friends has for women in relation to their feelings of pregnancy acceptability. The use of self-report questionnaires allowed us to collect a range of data from a large sample with minimal consumer and healthcare worker burden. However, reliance on self-report means that symptomatology may have been under or over-reported. Finally, we recognise that the cross-sectional design of the study offers only a snapshot view of pregnancy acceptability from a modest single community sample of women within an Australian context. Given we know women’s views about pregnancy may change from pre-conception and throughout pregnancy [4, 29], longitudinal research in this area would be valuable.

### Recommendations

Continued exploration of the pregnancy acceptability model as an adjunct to the pregnancy intendedness model is needed to determine its role as a potential indicator for women at risk for mental health and bonding difficulties. This approach is also consistent with a more holistic understanding of women’s wellbeing that is focused on individual experiences. Greater knowledge of the role of pregnancy acceptability in women’s experiences of pregnancy may assist health professionals to support women who would benefit from targeted interventions to improve outcomes for mother and baby.

## Conclusion

Findings from our sample of 116 Australian pregnant women provide the first evidence that pregnancy acceptability may not only be associated with women’s mental health and bonding during pregnancy, but that it may impact upon this relationship. The complex relationship between women’s mental health and antenatal bonding can be better understood when consideration is given to women’s individual characteristics and circumstances. This paper highlights that pregnancy acceptability may be an important factor in the way women feel about themselves and their baby, especially when they experience mixed or negative feelings towards their pregnancy.

## Data Availability

The datasets used and/or analysed during the current study are available from the corresponding author on reasonable request.
